# Serum leptin levels in patients with chronic kidney disease and hypertensive heart disease: An observational cross‐sectional study

**DOI:** 10.1002/hsr2.1053

**Published:** 2023-01-19

**Authors:** Emmanuel K. Ofori, Christian N. Adekena, Vincent Boima, Henry Asare‐Anane, Ernest Yorke, Eric N. Y. Nyarko, Bismark N. Mohammed, Emmanuel Quansah, Sisitha U. Jayasinghe, Seth D. Amanquah

**Affiliations:** ^1^ Department of Chemical Pathology University of Ghana Medical School Accra Ghana; ^2^ University of Ghana Medical Center Accra Ghana; ^3^ Department of Medicine and Therapeutics University of Ghana Medical School Accra Ghana; ^4^ School of Health Sciences University of Tasmania Hobart Tasmania Australia

**Keywords:** body mass index, chronic kidney disease, glomerular filtration rate, hypertension, leptin

## Abstract

**Background and Aim:**

Adipocytes secrete a peptide hormone called leptin, which plays a crucial role in controlling appetite and energy expenditure. Alterations in leptin concentrations are associated with CKD‐related cardiovascular problems such as hypertensive heart disease (HHD). Despite the link, data on the precise function of leptin in people with CKD and HHD is scant.

**Methods:**

An observational cross‐sectional study involving a total of 108 participants (72 CKD patients with HHD and 36 healthy controls). Their demographic and anthropometric information was collected using a standardized questionnaire. Certain clinical measures such as blood pressure and body mass index (BMI) were assessed. Fasting blood samples were analyzed for levels of plasma glucose (FPG), lipids, creatinine, and leptin. Data were analyzed with SPSS v23.

**Results:**

Leptin, FPG, creatinine and triglyceride levels were all significantly higher in CKD patients with HHD compared to controls (*p* < 0.01 for all). Furthermore, advanced CKD status (being in stage 5), having a 6‐year diagnosis of HHD, being female, having a higher BMI, and elevation in levels of HDL and FPG contributed significantly to the variance in serum leptin levels in the case group (*β* = 0.37, 0.22, 0.19, 0.18, 0.27, 0.28; *p* < 0.05 for all). In the control group, the female gender had the biggest unique effect on circulating leptin levels, followed by BMI and eGFR (*β* = 0.71, 0.34, −0.22; *p* < 0.01 for all).

**Conclusion:**

Patients with CKD who also had HHD reported considerably higher circulating leptin levels. Significantly higher blood leptin levels were shown to be associated with CKD stage 5 in the case group. These results are consistent with the role of leptin in the metabolic complexity seen in CKD patients. There needs to be more research into treatments that aim to lower leptin levels in CKD patients with HHD.

## 1 INTRODUCTION

Chronic kidney disease (CKD), is associated with a gradual decline in glomerular filtration rate (GFR). It is a debilitating condition that affects millions of people all over the world.^1^, ^2^, ^3^, ^4^ The ineffective management of risk factors for the vast majority of chronic nephropathies and the ongoing progression to end‐stage kidney disease (ESKD) both contributes to an increase in the incidence of CKD over the world.^4^, ^5^, ^6^

Major risk factors for developing CKD include diabetes mellitus, high blood pressure, and other cardiovascular‐associated diseases.^7^, ^8^ These risk factors can only explain a portion of the increased fatality rates in CKD patients. The last two decades have seen considerable technological and pharmacological CKD therapy advancements, although these innovations have not yet resulted in any substantial changes in CKD survival rates.^9^, ^10^ Sub‐Saharan African nations, including Ghana, have a particularly higher prevalence of CKD ranging from 5% to 17%.^5^, ^11^ The rising prevalence of CKD is a public health emergency in Ghana, despite a significant scarcity of pertinent knowledge regarding its epidemiology, prognosis, and treatment.^12^, ^13^ Amongst a myriad of reasons including low levels of public awareness, unpreparedness to cope with the cardiovascular consequences of CKD, and the lack of access to renal replacement therapy, are considered major contributors to the CKD epidemic in Ghana and other sub‐Saharan nations.^14^, ^15^

Hypertensive heart disease (HHD) is a group of changes in the left ventricle, left atrium, and coronary arteries caused by high blood pressure that lasts for a long time. It is linked to heart failure, ischemic heart disease, stroke, and deaths caused by hypertension.^16^, ^17^ HHD is characterized by anatomical and physiological changes in the myocardium in the absence of other primary anomalies in the cardiovascular system, resulting in a collection of responses from target organs ranging from systolic and diastolic dysfunctions to left ventricular hypertrophy (LVH) and their clinical manifestations (arrhythmias and heart failure).^18^, ^19^ CKD patients are highly susceptible to HHD.^20^, ^21^

Leptin is a peptide hormone that controls how much energy the body uses.^22^, ^23^ It is mostly made by adipocytes (mostly white adipocytes and less often brown adipocytes) and plays a big role in controlling the body's energy balance and reducing hunger.^24^, ^25^ Leptin in the bloodstream may be complexed with proteins or it may circulate freely.^26^, ^27^ Leptin is involved in a wide variety of different physiological processes in addition to its primary function of regulating the amount of fat stored in the body. These activities include bone formation, immunological function, angiogenesis, and fertility^28^, ^29^, ^30^ although the mechanics of these secondary actions remain to be fully elucidated. Accordingly, relatively little research has been conducted to investigate the link between elevated blood leptin levels and CKD, with the majority reporting higher than normal levels of leptin in CKD patients,^31^, ^32^ alongside a sleuth of cardiovascular complications.^33^, ^34^

Specifically, the interface between CKD, leptin, and HHD has received much less attention considering the enormity of the CKD epidemic. Given the significant economic burden on countries,^35^, ^36^, ^37^ and the negative health outcomes associated with leptin and CKD,^38^, ^39^, ^40^ as well as the role of both leptin and CKD in CVD mortality, more research into the role of leptin in CKD and HHD are merited. Therefore, the main purpose of this study is to report on levels of serum leptin and identify the characteristics that predispose CKD patients with HHD to have altered leptin concentrations in the blood.

## 2 MATERIALS AND METHODS

### 2.1 Study site, design participants

The Renal Dialysis Unit of the Korle‐Bu Teaching Hospital (KBTH) in Accra was the site where the study was carried out. Patients suffering from acute and chronic renal failure are under the care of the Unit's management. A total of 72 patients with CKD who also had HHD (i.e., had a documented diagnosis of HHD by a qualified specialist and/or were on chronic hemodialysis) and 36 healthy individuals (hospital staff) were recruited into this study using the simple random sampling technique. CKD patients were required to have an eGFR of <60 mls/min for more than 3 months, and/or have an ultrasound scan showing small kidneys measuring less than 9 cm. Individuals between the ages of 30 and 70 who did not have CKD, were not on admission to the hospital, or had been diagnosed with a specific condition for which they were receiving the medicine were considered to be healthy adults for this study and were included as controls. Patients with CKD who were undergoing transplantation, as well as those who had a history of *Mycobacterium tuberculosis*, autoimmune illness, gout, allergies, cancer, urinary tract infection, or acute cardiovascular events, were not allowed to participate in the study. A structured questionnaire was used to collect data on the participants, including their sociodemographics and clinical histories (Supporting Information file). The College of Health Sciences Ethical and Protocol Review Committee at the University of Ghana approved every step of the research process (CHS‐Et/M.3‐P2.7/2017–2018). Before being recruited for the study, participants were needed to fill out a consent form stating their willingness to participate. They were also informed of the following before consent: the duration of the study, the benefit of the study, the materials used in sample collection, and the potential risks involved with the sampling technique.

### 2.2 Clinical assessment and laboratory procedures

Participants' height (in m) and weight (in kg) were measured respectively with a calibrated wall‐mounted stadiometer (Secca) and a medical scale (Seca GmBh) using standardized procedures. To determine the participants' BMI, we divided their total body weight by the square of their height (kg/m^2^). An automated cuff blood pressure machine (OMRON) was used to take blood pressure readings of volunteers after they had sat still and relaxed for at least 10 min before the testing.

A trained phlebotomist collected venous blood (5 ml) from all participants after an overnight fast (8–12 h). One milliliter (ml) of blood was aliquoted into a sodium fluoride‐containing tube, and the resulting plasma was obtained for glucose measurement. The remaining 4 mls of whole blood were placed in serum separator tubes, spun, split into aliquots, and stored at −20°C until assayed. Fasting plasma glucose (FPG), total cholesterol (T. cholesterol), triglycerides (TG), high‐density lipoprotein (HDL) cholesterol, and creatinine concentrations were measured using the auto‐analyzer of the VITROS system (Ortho Clinical Diagnostics). To determine the amount of leptin, present in the sera, an enzyme‐linked immunosorbent assay (ELISA) was carried out in the solid phase (GenWay Biotech Inc). A previously established algorithm was utilized to calculate a value for the estimated glomerular filtration rate (eGFR).^41^

### 2.3 Statistical procedures

Data were analyzed with the Statistical Package for the Social Sciences (SPSS) version 23 (IBM SPSS Statistics for Windows, 2018). Descriptive statistics were applied to summarize the demographic and clinical characteristics of the participants. To analyze the differences in clinical and biochemical parameters that exist between the cases and the controls, an independent *t*‐test was carried out. Further, the magnitude of the observed effect was determined using the *η*2 test statistic. A multiple regression analysis was carried out to identify the features that make both groups more likely to have altered amounts of leptin in their blood. A preliminary investigation was carried out to establish whether or not the data exhibited any deviations from normality, linearity, residual independence, or homoscedasticity. *p* < 0.05 were considered statistically significant.

## 3 RESULTS

Seventy‐two CKD patients with HHD and 36 controls made up the total number of participants used in this study. Tables 1 and 2 reflect, respectively, the demographic and clinical (categorical) characteristics of the study participants. Approximately half of the participants in the case group were men (52.8%), and 45.8% of them had completed a postsecondary degree. Approximately 42% of the cases were in the fourth stage of the disease. Moreover, one‐third (37.5%) had suffered HHD for 1 to 3 years. Only a minority (9.7%) of the patients with CKD have been on dialysis for more than 3 years. Ischemic heart disease (IHD) was seen in the majority (44.4%) of CKD patients.

**Table 1:** Clinical features (categorical) of the study participants

Clinical features	CKD group	
	Number	%
CKD stage		
Stage 3A	7	9.7
Stage 3B	21	29.2
Stage 4	30	41.7
Stage 5	14	19.4
Period with CKD		
Less than 1 year	23	31.9
1–3 years	29	40.3
More than 3 years	20	27.8
Period on dialysis		
Nondialysis CKD	39	54.2
Less than 1 year	5	6.9
1–3 years	21	29.2
More than 3 years	7	9.7
HHD category		
LVH	20	27.8
CHF	20	27.8
IHD	32	44.4
Period with HHD		
Less than 1 year	21	29.2
1–3 years	27	37.5
Greater than 3–6 years	13	18.1
More than 6 years	11	15.3

Abbreviations: CHF, congestive heart failure; CKD, chronic kidney disease; HHD, hypertensive heart disease; IHD, ischemic heart disease; LVH, left ventricular hypertrophy.

**Table 2:** Sociodemography and clinical characteristics (categorical) of participants

Variables	CKD	Controls	Chi‐square	*p* Value
	*N* = 72 (%)	*N* = 36 (%)	(*χ* ^2^)	
Age (years)				
<50	31 (43.1)	24 (66.7)	5.354	0.021
≥50	41 (56.9)	12 (33.3)		
Gender				
Male	38 (52.8)	19 (52.8)	1.0 × 10^−^^7^	0.98
Female	34 (47.2)	17 (47.2)		
Educational status				
No formal education	1 (1.4)	0 (0.0)	7.487	0.058
Primary	13 (18.1)	2 (5.6)		
Secondary	25 (34.7)	8 (22.2)		
Tertiary	33 (45.8)	26 (72.2)		
Occupation				
Self‐employed	50 (69.4)	11 (30.6)	10.800	0.005
Gainfully employed	20 (27.8)	23 (63.9)		
Unemployed	2 (2.8)	2 (5.6)		
BMI (kg/m^2^)				
Normal weight (18–24.9)	30 (41.7)	33 (91.7)	24.750	<0.0001
Overweight (25.0–29.9)	39 (54.2)	3 (8.3)		
Obese (>30)	3 (4.2)	0 (0.0)		
Blood pressure				
Normotensive	4 (5.6)	36 (100.0)	91.800	<0.0001
Hypertensive	68 (94.4)	0 (0.0)		

*Note*: Data are presented as numbers (percent). Categorical variables were compared using the *χ* ^2^ test. *p* < 0.05 is considered statistically significant.

Abbreviations: CKD, chronic kidney disease; *χ* ^2^, Chi‐Square test.

The clinical (continuous) and biochemical characteristics of the patients in the study are presented in Table 3. Using a *t*‐test on independent samples, we found that the case group were older and had a higher BMI, blood pressure, FPG, total cholesterol, triglyceride, low‐density lipoprotein, creatinine, and leptin levels than the control group (*p* < 0.05 for all, two‐tailed). Unsurprisingly, the eGFR in the group with CKD and HHD was significantly lower than that of the control group (*p* < 0.05, two‐tailed).

**Table 3:** Comparison of clinical and biochemical parameters between cases and controls

Variables	CKD	Controls	*p* Value
Age	52.29 ± 12.98	43.83 ± 11.40	0.0012
BMI (Kg/m^2^)	25.35 ± 2.733	22.86 ± 1.046	<0.0001
Blood pressure			
SBP (mmHg)	162.3 ± 19.20	113.3 ± 4.889	<0.0001
DBP (mmHg)	99.90 ± 11.97	75.08 ± 3.729	<0.0001
Lipid profile			
T. Cholesterol (mmol/L)	6.104 ± 1.440	4.544 ± 0.581	<0.0001
Triglycerides (mmol/L)	1.900 ± 0.661	1.492 ± 0.439	0.0011
HDL‐cholesterol (mmol/L)	1.342 ± 0.189	1.467 ± 0.167	0.0011
LDL‐cholesterol (mmol/L)	3.894 ± 1.182	2.383 ± 0.447	<0.0001
VLDL (mmol/L)	0.868 ± 0.297	0.683 ± 0.201	0.0011
Renal function test			
Serum creatinine	292.8 ± 136.6	72.89 ± 10.39	<0.0001
eGFR	27.33 ± 12.12	117.1 ± 18.51	<0.0001
FBG (mmol/L)			
Both	6.276 ± 1.973	4.733 ± 0.638	<0.0001
Male	5.939 ± 1.644	4.647 ± 0.611	0.0017
Female	6.653 ± 2.252	4.829 ± 0.672	0.0021
Leptin (ng/ml)			
Both	25.42 ± 12.92	5.683 ± 3.929	<0.0001
Male	22.45 ± 11.78	2.500 ± 0.574	<0.0001
Female	28.96 ± 13.50	9.241 ± 2.814	<0.0001

*Note*: Data presented as mean ± standard deviation (SD). Continues variables compared using unpaired *t*‐test and *p* < 0.05 considered statistically significant.

Abbreviations: CKD, chronic kidney disease; DBP, diastolic blood pressure; SBP, systolic blood pressure; VLDL, very low‐density lipoprotein.

The results of the regression analysis conducted on the case group are presented in Table 4. The standard multiple regression model contained the following variables: CKD stage, HDL‐cholesterol, FPG, HHD category and diagnosis, blood pressure, years of CKD diagnosis, gender, BMI, LDL‐cholesterol, and triglycerides. The model was able to explain 79.2% of the variation in terms of the mean levels of leptin found in serum. Being at CKD stage 5 had the biggest contribution (*β* = 0.370, *p* < 0.001) after adjusting for confounding factors. Other factors that significantly contributed to the overall variation in mean serum leptin levels include HDL (*β* = 0.269, *p* < 0.001), FPG (*β* = 0.267, *p* < 0.01), HHD diagnosis of more than 6 years (*β* = −0.217, *p* < 0.05), systolic BP (*β* = 0.201, *p* < 0.05), female gender (*β* = 0.191, *p* < 0.01), BMI (*β* = 0.180, *p* < 0.05), and LDL (*β* = 0.177, *p* < 0.05).

**Table 4:** Predictors (regression analysis) of increasing blood leptin levels among the CKD group

Variables	Unstandard beta (*β*)	Standard beta (*β*)	95% CI for beta (*β*)	*p* Value
CKD stage				
CKD stage 4	ref			
CKD stage 5	12.98	0.370	8.12–17.83	0.0001
CKD stage 3A	0.58	0.130	−6.29–7.44	0.8570
CKD stage 3B	−0.25	−0.011	−4.84–4.33	0.9040
HDL‐cholesterol	19.86	0.269	10.37–29.34	0.0001
FBG	1.89	0.267	0.86–2.92	0.0012
Years of HHD diagnosis				
<1 year	ref			
1–3 years HHD diagnosis	−4.29	−0.150	−8.91–0.34	0.0690
4–6 years HHD diagnosis	−2.03	−0.056	−8.03–3.99	0.501
>6 years HHD diagnosis	−8.37	−0.217	−15.35–1.37	0.020
Systolic BP	0.15	0.201	0.20–0.27	0.0320
Diastolic BP	−0.09	−0.07	−0.29–0.15	0.3920
Female gender	5.31	0.191	1.61–9.00	0.0059
BMI	0.92	0.180	0.17–1.67	0.0170
LDL‐cholesterol	2.09	0.177	0.14–4.05	0.0368
Years on dialysis				
Nil	ref			
≤3	2.84	0.009	−3.62–9.29	0.3820
>3 years of dialysis	−2.62	−0.06	−11.53–6.30	0.5580
CHF	−3.86	−0.125	−8.89–1.16	0.1294
IHF	−3.37	−0.121	−7.68–0.94	0.372
Years CKD diagnosis				
<1	ref			
≤3 years CKD diagnosis	2.22	0.079	−2.47–6.91	0.346
>3 years CKD diagnosis	6.15	0.198	−0.72–12.99	0.078

*Note*: *N* = 72; *df* = 71; *F* = 13.898; *R* ^2^ = 0.854; Adj. *R* ^2^ = 0.792; *p* < 0.05 is considered significant.

Abbreviations: BMI, body mass index; BP, blood pressure; CHF, congestive heart failure; CKD, chronic kidney disease; FBG, fasting blood glucose; HHD, hypertensive heart disease; IHF, ischemic heart failure.

The findings of the regression analysis for the control group are outlined in Table 5. Female gender, age, BMI, blood pressure, FPG, TG, LDL, HDL, and eGFR were all included in the conventional multiple regression model. This model was able to explain 89.4% of the variance in the levels of leptin in the blood. Female gender contributed the most significantly (*β* = 0.709, *p* < 0.001), followed by BMI (*β* = 0.341, *p* < 0.001), and eGFR (*β* = −0.222, *p* < 0.05).

**Table 5:** Predictors (regression analysis) of increasing blood leptin levels among the control group

Variables	Unstandard beta (*β*)	Standard beta (*β*)	95% CI for *β*	*p* Value
Female gender	5.51	0.709	4.37–6.64	0.0001
BMI	1.28	0.341	0.70–1.86	0.0001
FBG	0.06	0.009	−0.85–0.97	0.899
eGFR	−0.22	−0.222	−0.08–0.02	0.012
Age	0.03	0.062	−0.04–0.08	0.414
Systolic BP	0.11	0.131	−0.02–0.23	0.076
Diastolic BP	0.01	0.003	−0.15–0.16	0.952
Triglyceride	−0.64	0.071	−2.22–0.94	0.404
LDL‐cholesterol	−1.16	−0.131	−2.61–0.30	0.114
HDL‐cholesterol	0.81	0.034	−2.76–4.37	0.636

*Note*: *N* = 36; *df* = 35; *F* = 30.398; *R* ^2^ = 0.924; Adj. *R* ^2^ = 0.894; *p* < 0.05 is considered significant.

Abbreviations: BMI, body mass index; BP, blood pressure; eGFR, estimated GFR; FBG, fasting blood glucose.

## 4 DISCUSSION

A primary goal of this research was to report on serum leptin levels in CKD patients with HHD and to find out some characteristics that predispose CKD patients with HHD to have altered leptin concentrations in their blood. We found that the cases (CKD patients with HHD) had considerably greater levels of leptin in their blood when compared to the control group (Table 3), which suggests that CKD is a key factor in boosting serum leptin levels. This was somewhat anticipated given the proclivity for poor renal clearance of blood leptin among CKD patients leading to the higher circulating leptin concentrations observed.^32^, ^42^, ^43^

Elevated levels of leptin can be associated with a range of metabolic complications. For instance, some studies have established a connection between elevated leptin concentrations and hyperglycemia, a rise in blood pressure, natriuresis, and renal dysfunction.^44^, ^45^ Indeed, elevations in levels of leptin have also been shown to enhance kidney endothelial cell proliferation, indicating a potential role in renal glomerulosclerosis.^46^, ^47^ Nevertheless, contradictory findings have been reported by Scholze et al.^48^ where lower serum leptin levels were associated with lower GFR among CKD patients on hemodialysis therapy. The research that Scholze and colleagues conducted in 2007 was a longitudinal study that lasted for 7 years. This is a considerable amount of time for the body to develop mechanisms that lower blood leptin levels. The research that was conducted by Wahba and Mak^49^ in 2007, which found that the expression of the gene that codes for leptin were reduced in the adipose tissues of patients with CKD, provides support for the existence of this potentially adaptive phenomenon.

All of the CKD patients in this research also had HHD. Evidence to date has shown that leptin is associated with the development of cardiovascular problems by increasing cellular enzymatic activities which in turn increases the proliferation, calcification, and migration of vascularized cells.^50^, ^51^ The regression model that took into account the predictor factors was able to explain 79.2% of the variation in serum leptin levels among those who had CKD and HHD. Importantly, the most significant addition to the model was being at CKD stage 5 which predicted the existence of increased blood leptin levels and accounted for 8.47% of the observed variation (Table 4). This result that being at CKD stage 5 is related to greater blood leptin levels may bestow a survival advantage on this cohort of CKD patients. High levels of leptin have been observed in other contexts to predict favorable outcomes in patients who are receiving hemodialysis.^48^ That said, however, it is difficult to conclude if identifying CKD stage 5 as a predictor of higher serum leptin levels is relevant for this population at this time because the information on which such an inference may be made is insufficient, necessitating additional studies to complete knowledge gaps.

It is noteworthy that the time elapsed since the diagnosis of HHD was a significant predictor of serum leptin in CKD patients. Thus, in this cohort of CKD patients, being diagnosed with HHD for more than 6 years was related to lower serum leptin levels. This finding collaborated with an earlier study in 207 women with normal, impaired, or type 2 diabetes where lower leptin levels predicted increased cardiovascular mortality during a 7‐year follow‐up duration.^52^ While HHD diagnosis in a CKD patient for more than 6 years may compensate for the aberrant kidney's reduced ability to take up leptin from circulation,^53^, ^54^ it is quite unclear whether such reductions in serum leptin levels could predict improved outcomes in CKD patients diagnosed with HHD, as proposed by Scholze et al.^48^ This is because HHD on its own is not connected with any favorable outcomes.^19^, ^55^

According to the findings of this study, high levels of HDL and LDL cholesterols, both of which have been connected with a variety of cardiovascular events,^56^, ^57^, ^58^ substantially predicted elevated levels of serum leptin in the case group, with each factor contributing 5.15% and 1.35% respectively to the overall variation observed (Table 4). Furthermore, this study identified elevated FPG as a significant predictor of increased serum leptin levels in patients with CKD and a risk factor for metabolic diseases.^59^, ^60^ The relationship between FPG and serum leptin has been looked at in prior studies with the latter exerting effects on the homeostasis of glucose and insulin, some of which were independent of whole‐body adiposity and of specific regions of the body.^61^, ^62^

Some studies have reported BMI^63^, ^64^ and female gender^65^, ^66^ as determinants of increasing serum leptin in health and disease states. According to the findings of this study, these two factors are reliable indicators of increased blood leptin levels not just in patients with CKD and HHD but also in persons who appear to be in good health. Furthermore, eGFR was shown to predict leptin levels among the control group and corroborated prior studies conducted in Japan and Taiwan.^67^, ^68^ On the other hand, an experimental early‐stage type 2 diabetic mouse model showed a correlation between elevated leptin levels and an increase in GFR,^69^ contrary to what was observed among our control cohort. It is important to mention that the majority of eGFR studies have focused on metabolic risk,^70^, ^71^ resistin,^72^, ^73^ adiponectin,^74^, ^75^ serum creatinine, serum cystatin C, and lean tissue mass.^76^, ^77^ In light of these findings, it is of the utmost importance to emphasize the requirement for more research into the association between GFR and serum leptin, in addition to advancements in methodologies for accurate GFR prediction.

The condition of multicollinearity was violated by the measured total cholesterol and triglycerides, and as a result, were not included in the regression model. This was done to avoid producing an overdispersion of the data, which would have put the reliability of the conclusions drawn from the data at risk. Therefore, the proportions that remained unexplained in these models may be accounted for by the missing components, in addition to additional variables that were not studied in this work.

In summary, individuals with CKD who also had HHD exhibited higher blood leptin levels. In the case group, the presence of CKD stage 5 and high HDL cholesterol showed as the greatest predictors of serum leptin. Within the control group, the greatest predictors of blood leptin levels were found to be female gender, high BMI, and eGFR. More research is needed to fully understand the consequences that elevated leptin levels in CKD patients might have.

This study has several drawbacks. A larger number of study participants could not be sampled due to the specific characteristics of the target research group including our inability to obtain a representative number of CKD patients without HHD, and HHD patients without CKD from the case population. Assaying of free forms of leptin was a limitation in the present study. Additionally, the eGFR of the control group may be greater than what was reported, as it has been shown that the MDRD equation provides an inaccurate prediction of GFR in individuals who appear to be healthy.^78^, ^79^ Furthermore, because this was a cross‐sectional investigation, we were restricted in determining the reason or cause for the observed relationships. It would be very helpful to have follow‐up data to determine whether or not our findings are the consequence of causal relationships. Because leptin plays an important role in renal illness, more study is required to ascertain the mechanistic and synergistic repercussions of identified relationships, as well as the influence of circulating leptin levels on metabolic health.

## AUTHOR CONTRIBUTIONS

**Emmanuel K. Ofori**: Conceptualization; supervision; writing – original draft; writing – review & editing. **Christian N. Adekena**: Formal analysis; investigation; writing – original draft. **Vincent Boima**: Conceptualization; supervision; writing – review & editing. **Henry Asare‐Anane**: Writing – review & editing. **Ernest Yorke**: Writing – review & editing. **Eric N. Y. Nyarko**: Writing – review & editing. **Bismark N. Mohamme**d: Investigation; methodology. **Emmanuel Quansah**: Investigation; methodology. **Sisitha U. Jayasinghe**: Writing – review & editing. **Seth D. Amanquah**: Supervision; writing – review & editing.

## CONFLICT OF INTEREST

The authors declare no conflict of interest.

## TRANSPARENCY STATEMENT

The lead author Emmanuel K. Ofori affirms that this manuscript is an honest, accurate, and transparent account of the study being reported; that no important aspects of the study have been omitted; and that any discrepancies from the study as planned (and, if relevant, registered) have been explained.

## Data Availability

The corresponding author will provide the interested party with access to the data sets that were used throughout this study upon reasonable request.
